# A Porcine Pneumothorax Model for Teaching Ultrasound Diagnostics

**DOI:** 10.1111/j.1553-2712.2012.01349.x

**Published:** 2012-05-17

**Authors:** Nils Petter Oveland, Erik Sloth, Gratien Andersen, Hans Morten Lossius

**Affiliations:** From the Department of Research and Development, Norwegian Air Ambulance Foundation (NPO, HML)Droebak, Norway; the Department of Anaesthesiology and Intensive Care, Stavanger University Hospital (NPO)Stavanger, Norway; the Institute of Clinical Medicine Aarhus University (ES)Aarhus, Denmark; the Department of Anaesthesiology and Intensive Care, Aarhus University Hospital Skejby (ES)Aarhus, Denmark; and the Department of Radiology, Aarhus University Hospital Skejby (GA)Aarhus, Denmark

## Abstract

**Objectives:** Ultrasound (US) is a sensitive diagnostic tool for detecting pneumothorax (PTX), but methods are needed to optimally teach this technique outside of direct patient care. In training and research settings, porcine PTX models are sometimes used, but the description of the PTX topography in these models is lacking. The study purpose was to define the distribution of air using the reference imaging standard computed tomography (CT), to see if pleural insufflation of air into a live anaesthetized pig truly imitates a PTX in an injured patient.

**Methods:** A unilateral catheter was inserted into one pleural cavity of each of 20 pigs, and 500 mL of air was insufflated. After a complete thoracic CT scan, the anterior, lateral, medial, basal, apical, and posterior components of the PTXs were compared. The amount of air in each location was quantified by measuring the distance from the lung edge to the chest wall (LE-CW). A supine anteroposterior chest radiograph (CXR) was taken from each model and interpreted by a senior radiologist, and the image results were compared to CT.

**Results:** All 20 hemithoraces with PTX were correctly identified by CT, while six remained occult after interpreting the CXRs. The PTXs were anterior (100%), lateral (95%), medial (80%), basal (60%), apical (45%), and posterior (15%). The major proportion of the insufflated 500-mL volume was found in the anterior, medial, and basal recesses.

**Conclusions:** The authors found the distribution of the intrathoracic air to be similar between a porcine model and that to be expected in human trauma patients, all having predominantly anterior PTX topographies. In a training facility, the model is easy to set up and can be scanned by the participants multiple times. To acquire the necessary skills to perform thoracic US examinations for PTX, the porcine models could be useful.

Pneumothorax (PTX) is common after blunt chest injury.^1,2^ Failure to diagnose and rapidly treat an enlarging PTX may cause patient death.^3^ The advancements in imaging technology have led to an increased emphasis on thoracic ultrasound (US), found to be more sensitive than and as specific as supine anteroposterior chest radiographs (CXRs) in diagnosing traumatic PTX.^4–6^ Computed tomography (CT) is the diagnostic reference standard, but the need to transfer the patients to the radiology department may result in delayed diagnosis and treatment.^7,8^

The necessary training requirements to accurately perform thoracic US examinations have never been elaborated.^9^ Especially in time-critical, irreproducible, and unstable trauma patient situations, teaching the complexity of thoracic US is difficult; therefore, experimental PTX models are necessary. A model must be able to demonstrate four dynamic US signs: the lung sliding, the B-lines, the lung point, and the lung pulse, all originating from the pleural interface.^10^ The technical skills to operate the US machine, visualize the pleural line, and systematically search for the US lung signs to diagnose a PTX are important. Cadavers have been used for this purpose because they are ideal anatomical models, but a major drawback is the absence of any heartbeat. This reduces the realism of the US scans, especially since the lung pulse sign is caused by transmission of heart beats through the lung parenchyma. At best, a cadaver model randomized to tracheal or esophageal intubation could be used to study the presence or absence of the lung sliding sign.^11,12^ The same can be achieved with a simpler experimental model using two intravenous pressure bags submerged in water,^13^ but none of these models actually look at a real PTX. In medical simulation, computer-operated manikins^14–16^ are used as pathologic models to facilitate training of both diagnostic and treatment algorithms. A Medline and Embase literature search (terms: pneumothorax *and* ultrasound *and* [simulators *or* manikins]) gave no results regarding the use of manikins with PTX for US skill training.

Live anesthetized pigs with induced PTXs do not have the shortcomings seen in the other models. Their respiratory and cardiovascular systems are very much like the human systems, making these animals an important resource in biomedical research.^17,18^ What is missing is a description of the PTX topography in this model. Two recent studies used pigs to teach their participants thoracic US, but none evaluated their experimentally induced PTX against other imaging capacities such as CXR or CT.^19,20^ In supine human trauma and critically ill patients, the topography of PTX is known.^21,22^ The intrathoracic distribution of air in porcine PTX models should be studied before these models are accepted into US educational programs and curriculums.

In this study, we examined an experimental porcine model meant for US diagnostic training and research. The purpose was to describe the PTX topography in these models using the reference imaging standard CT, to see if pleural insufflation of air into a live anesthetized pig truly imitates a PTX in an injured patient.

## Methods

### Study Design

This was a laboratory study of PTX in a porcine model. Qualified and experienced animal caretaker personnel monitored the health of the animals during the study period. The experiments complied with the guidelines for animal experimental studies issued by The Danish Inspectorate for Animal Experimentation under the Danish Ministry of Justice, which also approved the study, and adhered to the principles in the National Institutes of Health Guide for Care and Use of Laboratory Animals.^23^

### Animal Model

A total of 20 female Danish landrace pigs (mean ± SD body weight = 54.1 ± 4.9 kg) were used in this study. To create an experimental PTX, air was injected into the pleural space and was followed by a diagnostic evaluation with an anteroposterior CXR and a thoracic CT scan. The volumes of porcine and humans lungs are comparable, with a total lung capacity of 55 mL/kg.^18,24^ Five-hundred milliliters of injected air (9.3 mL/kg) make up one-sixth of the estimated mean total lung capacity of 3,000 mL and was chosen to create a PTX without a total lung collapse and pressure physiology (i.e., tension PTX). Furthermore, 500 mL of air will obliterate the lung sliding sign in a porcine model of this size (i.e., 4.3 mL/kg is sufficient),^19^ which is important if the model is to be used for US training. To determine which side received the PTX (right vs. left), coded envelopes were randomly drawn. The result was that eight pigs had a right PTX and 12 had a left PTX; none had a bilateral PTX.

### Study Protocol

**Figure 1:**
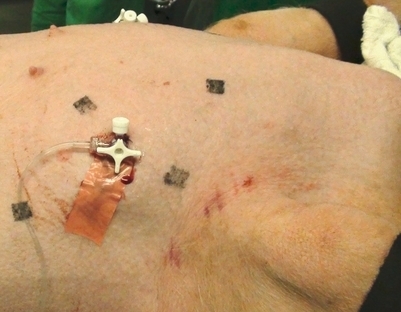
Porcine model with the catheter entering the pleural space.

Each animal was sedated, anesthetized, and intubated at the animal research laboratory. The anesthesia was maintained with a continuous infusion of fentanyl and propofol. A transport respirator (Oxylog 3000, Dräger Medical, Lübeck, Germany) was used and adjusted to a tidal volume of 11 to 15 mL/kg, a respiratory rate of 10 to 12 breaths/min, a positive end expiratory pressure of 2 to 4 cm H_2_O, and an inspiratory oxygen fraction of 30%, to keep the end-tidal carbon dioxide level within the normal range (4 to 6.5 KPa). All animals were monitored with electrocardiogram, core temperature, invasive arterial blood pressure, oxygen saturation, and end-tidal carbon dioxide level. The hair on the animals’ chests was removed using an electrical shaver, and a 10-cm three-way stopcock catheter (BD Connecta, BD Medical, Franklin Lakes, NJ) was inserted into the pleural space through a small thoracotomy at the crossing of the fifth to seventh intercostals and anterior axillary line (Figure 1). This catheter was chosen because it was invisible on the CXRs. The surgical incision was closed by subcutaneous and cutaneous stitches. The PTX in each pig was created by 10 consecutive insufflations of air over 1 minute using a 50-mL syringe (Omnifix, B. Braun Medical, Melsungen, Germany) connected to the catheter. The three-way stopcock catheter was closed after each injection so no air escaped and the syringe refilled with air. At the radiology department, the animals were fixed in the supine position on a CT table. At the conclusion of data collection, each animal was euthanized with an injection of phenobarbital.

### Diagnostic Tests

**Figure 2:**
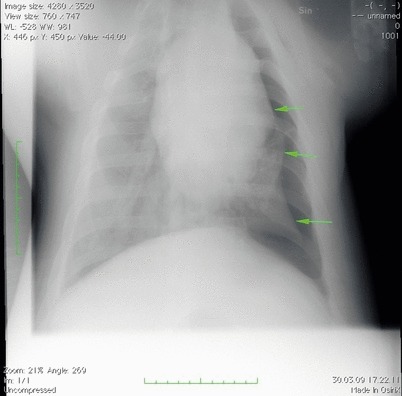
Supine anteroposterior CXR from the porcine model showing a left-sided PTX with readily apparent visceral pleural line without distal lung markings (*arrows*). CXR = chest radiograph; PTX = pneumothorax.

A supine anteroposterior CXR was obtained from each animal using a portable x-ray machine (Siemens Mobilett II, Siemens, Munich, Germany). An x-ray plate (Eastman Kodak, Rochester, NY) was placed underneath the thorax. The x-ray tube of the machine was brought into position 1 meter above the sternum. The radiographic field was adjusted and focused to include the thorax from the apex to the base. The radiograph was taken using standard imaging output adjustments (117 kV/1.25 mA), developed, and digitally stored. A senior radiologist, unaware of the CT image results, was asked to determine the presence or absence of a PTX in each CXR. The radiologist was preinformed that the radiographs could be normal or have a PTX in the left, right, or both hemithoraces. The CXRs were given encrypted names and presented to the radiologist in a randomized sequence by drawing a number and assigning it to each radiograph. The radiologic definition of PTX used was a readily apparent visceral pleural line without distal lung markings (Figure 2).^25^ The radiographs were viewed using a DICOM viewer (Philips R 2.6, Philips Medical Systems, Amsterdam, the Netherlands), and the PTX diagnoses were scored on a five-point scale (1 = definitely absent, 2 = probably absent, 3 = possibly present, 4 = probably present, and 5 = definitely present).^26^ When calculating the sensitivity and specificity, the five-point scale was dichotomized with a score of 4 or 5 being considered positive for PTX.

**Figure 3:**
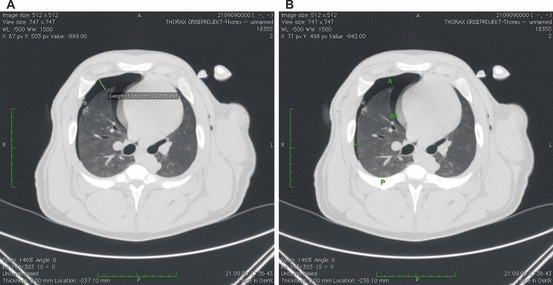
CT scan of a PTX, (A) Measurement of the LE-CW distance. (B) Intrathoracic distribution of the PTX. Air is in the anterior, medial, and lateral locations with no air posterior to the lung parenchyma. CT = computed tomography; LE-CW = lung edge–chest wall; PTX = pneumothorax.

To define the intrathoracic location of the air, a non–contrast-enhanced CT scan was performed using a multislice CT scanner (Philips MX 8000 quad, Philips Medical Systems, Best, the Netherlands) with the following parameters: 120 kV, 120 to 150 mA, standard filter, 6.5-mm slice thickness, 3.2-mm slice increment, and 310- to 360-mm field of view. A complete thoracic CT was obtained from the apex to the base during a short time period with inspiratory hold. The pictures were digitally stored and transferred to a picture archiving workstation. The window width was adjusted to 1500 Hounsfield units, and the window level to -500 Hounsfield units, to optimize the detection of the intrathoracic air. A second senior radiologist then analyzed and described the PTX topography according to the anterior, lateral, medial, basal, apical, or posterior position of the air relative to the lung parenchyma. As a semiquantitative measurement of the amount of air in each of these anatomic locations, the same radiologist measured the distance from the lung edge to the chest wall (LE-CW; Figure 3). This method is derived from an objective scoring system for occult PTXs, measuring the largest dimension of the largest air collection along a line perpendicular to the chest wall.^27^ A mean size of >7 mm is a potential factor associated with failure of observation and need of chest drain.^28^ Furthermore, the British Thoracic Society guidelines divides the size of a PTX into “small” or “large” depending on the presence of a visible rim of air less <20 mm or =20 mm between the lung margin and chest wall on an anteroposterior CXR.^29^ One measurement from each of the six anatomic positions was performed in each animal. The measurement was done at the CT slice level where the LE-CW distance was at its maximum, as estimated by the radiologist. Some locations did not contain any air and the distance was set to zero. The radiologist also went through the consecutive CT slices from each pig to assess any displacement of the mediastinal structures.

### Data Analysis

The continuous variable LE-CW distance was measured in millimeters. The mean distance with standard deviation (SD) per anatomic location (anterior, lateral, medial, basal, apical, posterior) was calculated based on the measurements from all of the 20 animals. The data were found to be normally distributed after performing the Shapiro-Wilk test (p = 0.05 in all six anatomic positions). The statistical power was low because of the small number of measurements (i.e., only three in the posterior position). The median, 25th percentile, 75th percentile, and interquartile ranges (IQRs) were therefore also reported. Furthermore, the number of occult PTXs in the 20 animals was determined comparing the result of each CXR to the reference standard CT. The CXR and CT data were incorporated into 2 × 2 frequency tables and the sensitivity, specificity, and their 95% confidence intervals (CI) were calculated.^30^ All the statistical calculations were performed using SPSS V 18.0 (IBM SPSS, Armonk, NY) and VasserStats (http://faculty.vassar.edu/lowry/VassarStats.html; Vassar College, Poughkeepsie, NY).

## Results

A total of 20 supine anteroposterior CXRs were obtained, but because two radiographs appeared blank after development, 18 valid hemithoraces with PTX, and 18 without were included. The radiologist correctly identified 12 hemithoraces (66.7%) with PTX, while six (33.3%) were falsely considered to be negative. The sensitivity of the supine anteroposterior CXR for detecting these PTXs was 66.7%, and the specificity was 83.3% (Table 1).

**1:** Sensitivity and Specificity of CXR and CT

Study	TP	FP	TN	FN	n*	*n*†	*n*‡	Sensitivity, %	95% CI	Specificity, %	95% CI
CT	20	0	20	0	40	0	40	100.0	80.0–100.0	100.0	80.0–100.0
CXR	12	3	15	6	36	4	40	66.7	41.2–85.6	83.3	57.7–95.6

CI = binomial confidence interval, calculated by use of normal approximation interval.^30^

CT = computed tomography; CXR = chest radiography; FN = false negative; FP = false positive; TN = true negative; TP = true positive.

* *n* = valid hemithoraces.

† *n* = missing hemithoraces.

‡ *n* = total hemithoraces.

**Figure 4:**
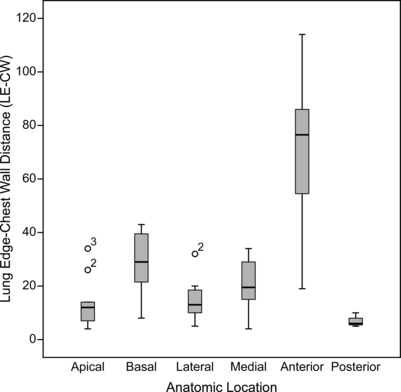
Box plot of the distance in the six anatomical positions. The bolded line inside the box represents the median value while the lower and upper limits of the box are the 25th and 75th percentiles. The vertical lines extend to the maximum and minimum values. The points above the box in the apical and lateral location are outliers and the numbers indicate the corresponding measurement. LE-CW = lung edge–chest wall.

The CT scans correctly identified all 20 hemithoraces with PTX and had no false positives, giving a sensitivity and specificity of 100% (Table 1). The intrathoracic anatomical locations of the air were anterior (100%), lateral (95%), medial (80%), basal (60%), apical (45%), and posterior (15%; Table 2). The amount of air in these locations varied between each porcine model. The rim of air was large (i.e., a mean LE-CW distance = 20 mm) in the anterior, basal, and medial parts of the pleural space, indicating a larger volume of air there compared to the smaller rim of air (i.e., a mean LE-CW distance < 20 mm) found in the lateral and apical locations, with almost no air behind the lung parenchyma (Table 2 and Figure 4). The 500 mL of insufflated air caused moderate displacement of the mediastinal structures in 55%, minor displacement in 30%, and none in 15% of the animals.

**2:** Distribution of Intrathoracic Air in a Porcine Model

Anatomic Location	Porcine PTX Distribution, *n* (%)	LE-CW Distance (mm)
			Mean	SD	SE mean	Median	25th percentile	75th percentile	IQR
Apical	9	45	13.9	10.0	3.3	12.0	7.0	14.0	7.0
Basal	12	60	29.5	11.0	3.2	29.0	21.5	39.5	18.0
Lateral	19	95	14.2	6.5	1.5	13.0	10.0	19.0	9.0
Medial	16	80	20.6	9.0	2.3	19.5	15.0	29.0	14.0
Anterior	20	100	69.1	27.5	6.1	76.5	54.5	86.0	31.5
Posterior	3	15	7.0	2.6	1.5	6.0	*	*	*

IQR = interquartile range between the 25th and 75th percentile; *n* = numbers of hemithoraces with air in the corresponding anatomical location; PTX = pneumothorax; SD = standard deviation; SE mean = standard error of mean.

* 25th percentile, 75th percentile, and IQR not calculated because of too few measurements.

## Discussion

Our study confirms that in a supine porcine model, all of the experimentally induced PTXs resulted in air distribution in the anterior location. We also found that the air is common in the medial (80%) and basal pleural spaces (60%), with little or no air escaping behind the lung. This is similar to what would be expected in a supine patient with no pleural adhesions or interfering thoracic injuries, where the intrathoracic air collects in the least-dependent anteromedial space and seldom in the posterior recess.^21,31^ The same distribution of air was found in nearly all of our models, confirming a close similarity between human and porcine PTX topography. The porcine lung has two lobes on the left side and four lobes on the right side,^18^ but this minor anatomic difference from human lungs did not alter the distribution. In humans the location of the intrathoracic air is directly associated with the force of gravity, the elastic recoil of the lung and chest wall, and the attachment of the lung to the hilar structures.^31,32^ This seems also to be true in a supine porcine model, since the air, if free to move, collects in the same anatomical positions. Among the distribution differences are an increased proportion of lateral PTXs in the models (95%). This effect may be explained by the more cone-shaped porcine thorax, which more easily allows the air to collect on the outside of the lung. The small and distinct shape of the porcine apical thorax could also explain why less air was found there. Despite some differences, the deviation from human trauma patients is arguably minor when analyzing the amount of air in each anatomical location. Most of the insufflated air is found in the anterior, medial, and basal recesses in our models (Figure 4), the same locations where air most commonly collects in humans.^31^

In the supine position, the anteroposterior CXR of the pigs failed to detect one-third of the PTXs, again comparable to radiographic studies of chest trauma patients.^4,33^ All of these occult PTXs were subsequently found on the CT scans. Although air in the anteromedial location is difficult to detect and quantify on a supine anteroposterior CXR, the anterior chest is one of the positions to be scanned with US in the extended focused assessment with sonography for trauma (e-FAST) protocol.^6^ The predominantly anterior PTX topography found in our models therefore makes this an ideal training model for US diagnosis of PTX. This is because an US probe placed on the anterior chest would likely be positioned over an area with the PTX.

The other advantages with porcine PTX models are that they can be used for multiple scans and are fairly easy to set up in a training facility. The model is universal and can be used by various medical specialties, all of whom may benefit from training before using thoracic US to detect PTX in trauma patients. The minimum number of thoracic US examinations necessary to achieve competency in this field is unknown,^9^ but the guidelines from the American College of Emergency Physicians suggest that trainees should perform between 25 to 50 examinations.^34^ In the European Federation of Societies for Ultrasound in Medicine and Biology (EFSUMB) guidelines, a minimum of 200 examinations are recommended to achieve a skill level 1 in thoracic US, encompassing multiple pathologic conditions such as pleural effusions, pericardial effusions, chest wall and lung diseases, and PTX.^35^ Several of these pathologic conditions could be induced in the same porcine model.^36,37^ US used on experimental models could supplement theoretical lectures and scanning of normal human subjects to acquire these necessary skills.

In the global perspective, two-thirds of the world’s population has no access to imaging technologies, while basic x-ray and US examinations potentially could solve up to 80% to 90% of all diagnostic problems.^38^ The importance of implementing US and developing effective curricula to teach health care providers how to use this technology could have wide-ranging implications. This could justify the future use porcine PTX models designed for US training and research.

## Limitations

This study has certain limitations. First, the porcine anatomy is not identical to that of humans; the thorax is more cone-shaped and this affects the intrathoracic distribution of air. The miss rate of PTX on the CXRs could be in part affected by this difference, but no previous studies on pigs discussing this exist. Furthermore, the radiologist was not blinded to the study purpose and was specifically told to look for PTXs in the pictures. This could improve the CXR diagnostic accuracy results, but the sensitivity and specificity were not higher than human radiographic studies. Second, the small thoracotomy may have introduced small amounts of air into the pleural cavity, thereby making the actual intrathoracic volume higher than the insufflated 500 mL of air.

A third point deserves to be underlined. Our porcine models were intubated and mechanically ventilated, which could affect the size and evolution of the PTX. However, in humans both the PTX size and the intrathoracic distribution of air in an intubated cohort have been reported to be similar to those of a nonintubated cohort,^21^ suggesting that PTX topography is not greatly affected by applying positive pressure ventilation. The use of our porcine models for US training should therefore be generalizable to human trauma patients, intubated or not. Last, the PTXs were not created in the normal pathophysiologic manner, but rather by insufflating air through an artificially placed tube. The placement of the catheter was not random, but at the same position of the chest in each animal. This may have affected the intrathoracic distribution of air, but did not result in any major differences in PTX topography compared to humans. The use of a different insufflation technique may not give the same result.

## Conclusions

We provide a detailed description of a porcine pneumothorax model that was easy to create with air distribution that closely mimics human pneumothorax and should be considered as a useful tool to teach ultrasound diagnostics. Additional studies on the use of US with this model are under way.
